# Submucosal tunneling endoscopic resection in challenging esophageal subepithelial lesions: a minimally invasive approach

**DOI:** 10.1016/j.igie.2025.10.006

**Published:** 2025-10-15

**Authors:** Shaimaa Elkholy, Hussein Okasha, Abeer Awad, Hany Haggag, Karim Essam, Kerolis Yousef, Mohamed Abdel Zaher, Mohamed El Sherbiny

**Affiliations:** 1 Gastroenterology Division, Internal Medicine Department, Faculty of Medicine, Cairo University, Cairo, Egypt; 2 Faculty of Medicine, Cairo University, Cairo, Egypt

## Abstract

**Background and Aims:**

Submucosal tunneling endoscopic resection (STER) is a minimally invasive procedure for resecting esophageal subepithelial lesions (SELs), particularly those arising from the muscularis propria layer. This article presents 2 challenging cases of esophageal SELs successfully managed with STER.

**Methods:**

Two esophageal SELs were managed with STER, which involved creating a submucosal tunnel for en bloc resection, followed by closure with hemostatic clips. Challenges related to lesion location, size, and patient comorbidities were addressed during the procedures.

**Results:**

Both patients underwent successful en bloc resection of the SELs without adverse events. One presented with progressive dysphagia for 2 years, and the other with dysphagia and intermittent chest pain. Both experienced significant clinical improvement postoperatively.

**Conclusions:**

STER offers an effective, minimally invasive alternative to surgery in challenging cases of esophageal SELs, demonstrating excellent results with reduced morbidity when performed with careful planning and multidisciplinary collaboration.

## Introduction

Submucosal tunneling endoscopic resection (STER) is an advanced endoscopic technique used for the removal of esophageal subepithelial lesions (SELs). The American Society for Gastrointestinal Endoscopy classifies endoscopic full-thickness resection into exposed and nonexposed types relative to the peritoneal cavity. The exposed category is further subdivided into tunneled and nontunneled approaches, with STER representing the tunneled variant. It allows en bloc resection of the lesion while preserving surrounding tissues and avoiding damage to adjacent structures.^1^ The procedure is categorized under exposed endoscopic full-thickness resection and is considered part of third-space endoscopy, where the mucosa serves as a protective flap.^2^

STER is particularly valuable in challenging cases where the lesion is close to vital structures such as the aortic arch or mediastinum, where traditional resection methods may pose a high risk.^3^ The primary goal of STER is to provide a minimally invasive alternative to surgery, offering a safe and effective means of lesion excision with reduced recovery time and morbidity.^3^ This manuscript describes 2 complex clinical cases performed as part of routine patient care.

## Methods

The STER method involves several critical steps to safely and effectively remove lesions. First, a mucosal incision is made approximately 3 to 5 cm proximal to the lesion, and a submucosal tunnel is carefully created, extending to the lesion site. This tunnel provides access to the lesion while minimizing damage to surrounding tissues. Next, the lesion is meticulously dissected from the surrounding structures within the tunnel, ensuring en bloc resection, which involves removing the lesion in 1 piece to preserve tissue integrity and minimize trauma. Finally, after successful removal of the lesion, the mucosal entry point is closed using hemostatic clips to prevent leakage, control bleeding, and promote proper healing. These steps collectively ensure the success and safety of the procedure.^4^^,^^5^

## Case presentations

Two cases illustrate the application of STER in challenging esophageal SELs.

### Case 1

**Figure 1:**
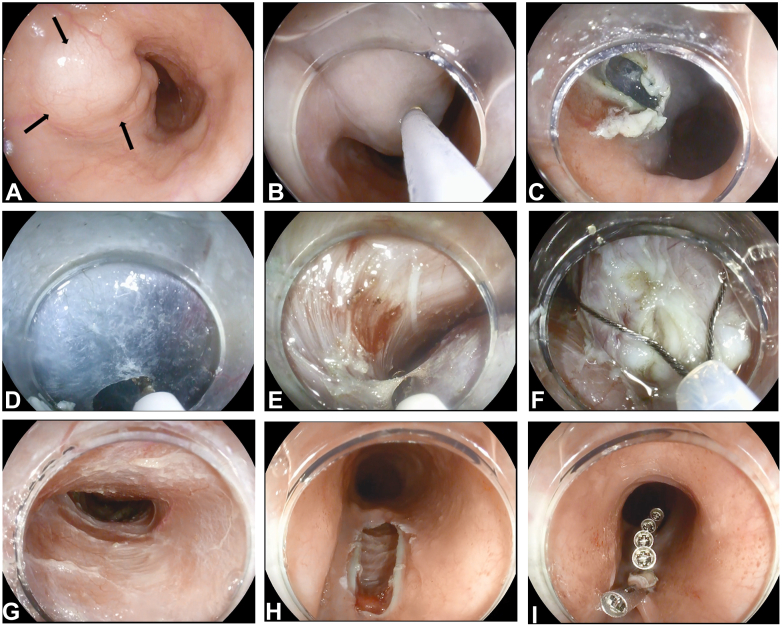
**A,** Endoscopic view of a large esophageal subepithelial lesion (*black arrows* indicating the tumor). **B,** Submucosal injection with diluted methylene blue. **C,** Mucosal incision to create the tunnel opening. **D and E,** Submucosal tunneling and dissection of the subepithelial tumor from surrounding tissues. **F,** Tumor extraction from the tunnel using a snare. **G,** Empty tunnel after extraction of the lesion. **H,** Wide-gaping mucosal incision visualized after removal of the tumor. **I,** Closure of the tunnel opening with endoscopic clips.

**Figure 2:**
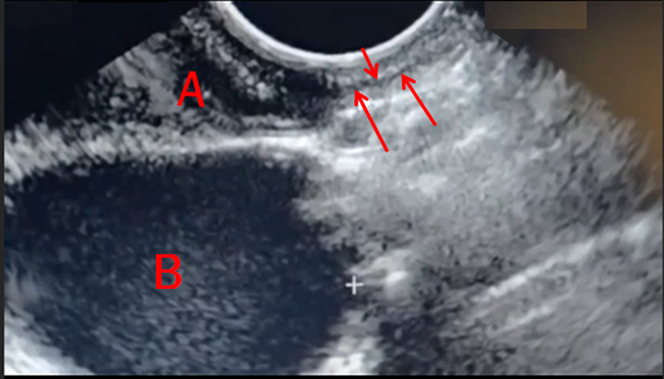
Endoscopic ultrasonography image showing a subepithelial lesion (**A**) originating from the fourth layer, specifically the circular muscle layer of the muscularis propria (indicated by *arrows*). The lesion is in close proximity (only 1 mm) to the aorta (**B**), posing a significant challenge because of the patient's comorbidities and the lesion's size.

**Figure 3:**
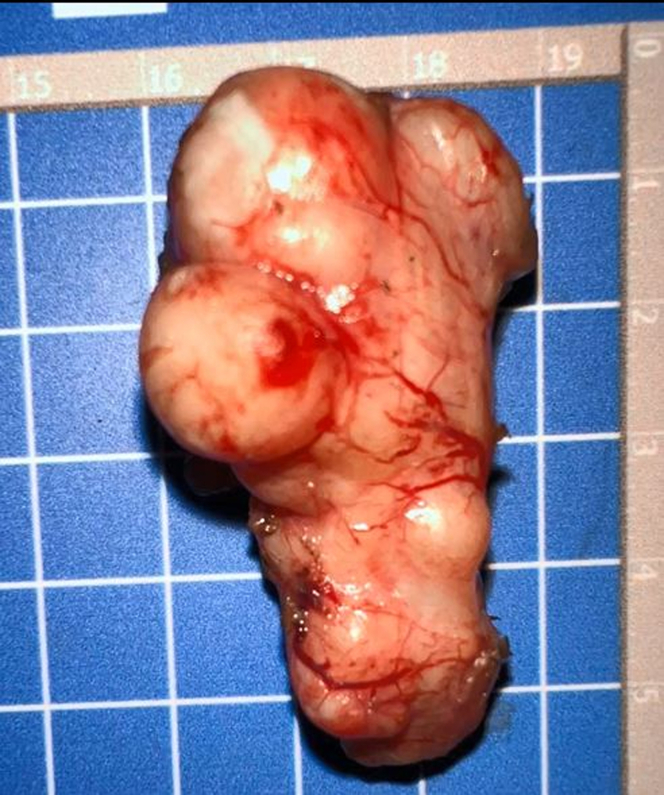
Endoscopic view of the retrieved large esophageal leiomyoma.

**Figure 4:**
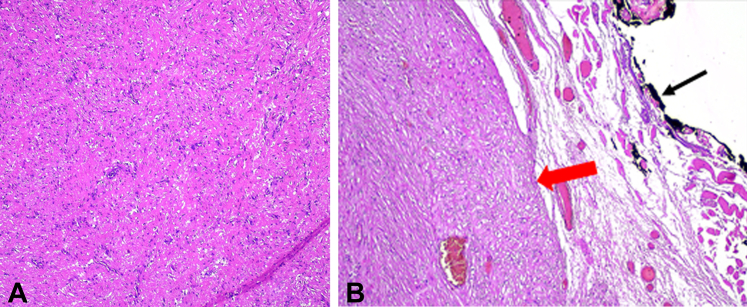
Histopathology of the first esophageal subepithelial lesion. **A,** H&E stain, original magnification ×100. Well-circumscribed spindle cell tumor with no necrosis or mitotic activity, consistent with leiomyoma. **B,** H&E stain, original magnification ×40. Inked deep margin (*black arrows*), confirming complete en bloc excision with negative margins. The *red arrow* delineates the edge of the leiomyoma. *H&E*, Hematoxylin and eosin.

A middle-aged woman with a history of systemic lupus erythematosus and complicated Crohn's disease presented with progressive dysphagia. Imaging revealed a large midesophageal SEL (6 × 3 cm) (Fig. 1), confirmed by endoscopic ultrasonography to originate from the circular muscle layer (Fig. 2). Because of the lesion's proximity to the aortic arch, traditional resection methods were deemed too risky. After a multidisciplinary consultation, STER was selected. A submucosal tunnel was created proximally to the lesion, and dissection was carefully performed to isolate the lesion. Selective myotomy was carried out on the circular muscle layer, preserving the longitudinal layer. The lesion was excised en bloc (Fig. 3), retrieved for histopathology, and the tunnel was closed with hemostatic clips. The patient's dysphagia markedly improved after the procedure, and she was able to resume oral fluid intake without difficulty. Pathologic analysis confirmed the lesion to be a leiomyoma, fully excised (Fig. 4) with no adverse events.^6^

### Case 2

**Figure 5:**
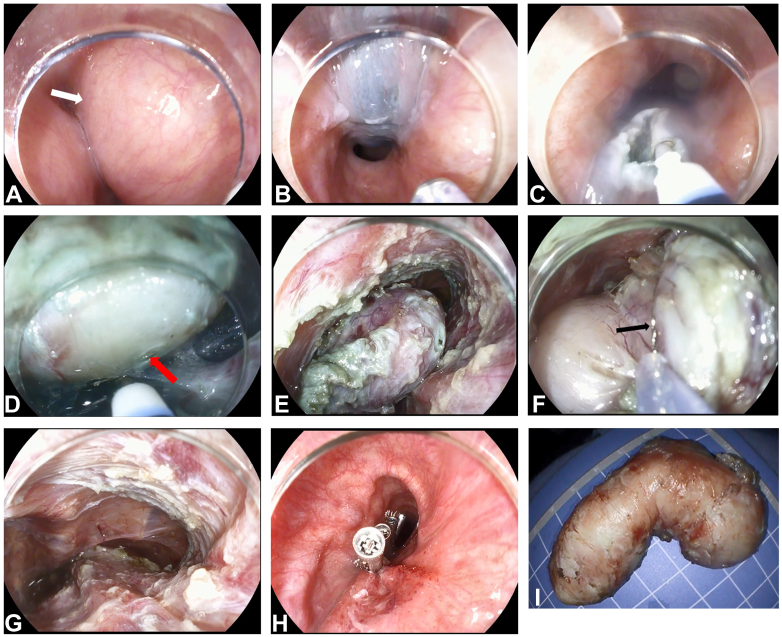
Endoscopic steps of submucosal tunneling endoscopic resection in case 2. **A,** Endoscopic view showing a large subepithelial lesion (*white arrow*). **B,** Submucosal injection with diluted methylene blue. **C,** Mucosal incision to create the tunnel opening. **D,** Submucosal dissection of the lesion from surrounding tissues (*red arrow* pointing to the lesion). **E,** Tumor inside the tunnel. **F,** Snare holding the lesion inside the tunnel prior to extraction (*black arrow*). **G,** Empty tunnel following lesion extraction. **H,** Closure of the tunnel entry using endoscopic clips. **I,** Gross specimen showing a well-encapsulated lesion with a smooth outer surface.

**Figure 6:**
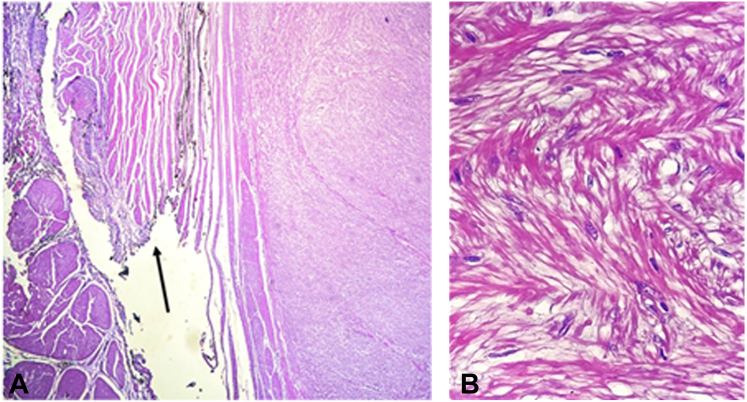
Histopathology of the second esophageal subepithelial lesion. **A,** H&E stain, original magnification ×40. Well-demarcated lesion with free inked margin (*black arrow*), confirming complete excision down to the muscularis propria. The lesion is located to the right of the *arrow*. **B,** H&E stain, original magnification ×100. Bland spindle cells with no necrosis or mitotic activity, consistent with leiomyoma. *H&E*, Hematoxylin and eosin.

A 42-year-old male patient with progressive dysphagia and chest pain was diagnosed with a large esophageal SEL (8 × 5 cm), originating from the muscularis propria layer. The lesion extended across both the anterior and left walls of the upper and middle esophagus, located approximately 22 cm from the incisors. His medical history included hypertension. Because of its size and location, the lesion restricted scope maneuverability and posed a higher risk of mediastinal exposure. Despite these challenges, STER was performed successfully. A submucosal tunnel was created as proximally as possible, and dissection was done to separate the lesion from the mucosa. Full-thickness myotomy was required to isolate the lesion, leading to mediastinal exposure, which was safely managed using an insulated-tip knife (Fig. 5). Retrieving this huge lesion en bloc was particularly challenging. To remove the lesion, steps included 1 operator holding the gastroscope and firmly grasping the snare catheter while the other endoscopist pulled the scope out. Anesthesia deflated the endotracheal tube cuff, and the patient's head was fixed in position. By maneuvering the gastroscope back and forth, the lesion was retrieved in 1 piece. Pathology confirmed a leiomyoma (Fig. 6), and the patient reported significant symptom relief after the procedure.^7^

## Discussion

STER is a promising, minimally invasive technique for the resection of complex esophageal SELs. Both cases demonstrated the utility of STER in challenging scenarios, including large lesion size and proximity to vital structures such as the aortic arch and mediastinum. By creating a submucosal tunnel and using careful dissection, STER enables en bloc resection of the lesion while minimizing injury to surrounding tissues. These findings are consistent with previously reported feasibility and safety of STER for upper gastrointestinal SELs.^8^ Furthermore, current international guidelines support the use of STER as a viable alternative to surgery in select cases.^9^ Multidisciplinary collaboration, along with meticulous procedural planning, ensures the success of this approach, making it an effective alternative to surgery with reduced morbidity.

Both cases presented unique anatomic and clinical challenges that highlight the advanced utility of STER beyond standard indications. In case 1, the lesion was located only 1 mm from the aortic arch in a patient with complex systemic comorbidities, including longstanding systemic lupus erythematosus and complicated Crohn's disease. The patient had a history of total colectomy and small-bowel resection, leaving only 1 meter of jejunum, and was partially dependent on total parenteral nutrition, which further increased her overall procedural risk. In case 2, the lesion was unusually large (8 × 5 cm), extending across the anterior and left walls of the upper and middle esophagus. Its location limited scope maneuverability and required full-thickness myotomy in close proximity to the mediastinum. These factors demanded tailored dissection strategies, careful risk mitigation, and multidisciplinary decision making, emphasizing STER's versatility in high-risk settings. Notably, both lesions were resected completely en bloc, despite their large size and challenging locations, further demonstrating the efficacy and safety of STER in such complex scenarios. These 2 cases demonstrate the potential of STER as a minimally invasive alternative to surgery for complex esophageal SELs, with favorable outcomes achieved through careful planning and technique.^10^

Nevertheless, our report has inherent limitations. It describes only 2 cases, limiting generalizability. The successful outcomes presented may not be reproducible in all settings, as STER requires advanced endoscopic skills and careful multidisciplinary planning. Additionally, performing STER in large lesions adjacent to vital structures such as the aorta or mediastinum increases the risk of adverse events, including perforation or mediastinal emphysema, as previously reported in the literature.^4^ These factors must be carefully weighed when considering STER in anatomically complex cases. Still, these cases illustrate how STER can be safely adapted to complex clinical and anatomic scenarios, potentially broadening its therapeutic scope in managing high-risk esophageal SELs.

## Patient Consent

Written informed consent was obtained from both patients prior to the procedures. In accordance with international ethical standards and institutional policies—including guidelines from the International Committee of Medical Journal Editors and the Committee on Publication Ethics—formal Institutional Review Board or ethics committee approval was not required.

## Disclosure

All authors disclosed no financial relationships.

## Supplementary data

Video 1 Here we present submucosal tunneling endoscopic resection (STER) as a minimally invasive approach for the management of challenging esophageal subepithelial lesions (SELs). Case 1: A 46-year-old woman with history of systemic lupus erythematosus, complicated Crohn disease, and progressive dysphagia over 2 years had esophagogastroduodenoscopy revealing a large 6 × 3-cm subepithelial lesion. EUS confirmed its origin from the muscularis propria and proximity to the aortic arch. After multidisciplinary consultation, STER was selected. Case 2: A 42-year-old man with progressive dysphagia and chest pain had a large 8 × 5-cm esophageal subepithelial lesion arising from the muscularis propria, extending across the anterior and left esophageal walls. Because of its size and location, STER was performed with full-thickness myotomy and successful en bloc removal.
